# *Dictyostelium* Differentiation-Inducing Factor-1 Promotes Glucose Uptake, at Least in Part, via an AMPK-Dependent Pathway in Mouse 3T3-L1 Cells

**DOI:** 10.3390/ijms22052293

**Published:** 2021-02-25

**Authors:** Yuzuru Kubohara, Yoshimi Homma, Hiroshi Shibata, Yoshiteru Oshima, Haruhisa Kikuchi

**Affiliations:** 1 Laboratory of Health and Life Science, Graduate School of Health and Sports Science, Juntendo University, Inzai, Chiba 270-1695, Japan; 2 Department of Biomolecular Science, Institute of Biomedical Sciences, School of Medicine, Fukushima Medical University, Fukushima 960-1295, Japan; yoshihom@fmu.ac.jp; 3 Laboratory of Epigenetics and Metabolism, Institute for Molecular and Cellular Regulation, Gunma University, Gunma 371-8512, Japan; hshibata@gunma-u.ac.jp; 4 Laboratory of Natural Product Chemistry, Graduate School of Pharmaceutical Sciences, Tohoku University, 6-3 Aza-Aoba, Aramaki, Aoba-ku, Sendai 980-8578, Japan; oshima@mail.pharm.tohoku.ac.jp (Y.O.); hal@mail.pharm.tohoku.ac.jp (H.K.)

**Keywords:** *Dictyostelium discoideum*, DIF-1, obesity, diabetes, AMP kinase

## Abstract

Differentiation-inducing factor-1 (DIF-1) is a chlorinated alkylphenone (a polyketide) found in the cellular slime mold *Dictyostelium discoideum*. DIF-1 and its derivative, DIF-1(3M) promote glucose consumption in vitro in mammalian cells and in vivo in diabetic rats; they are expected to be the leading antiobesity and antidiabetes compounds. In this study, we investigated the mechanisms underlying the actions of DIF-1 and DIF-1(3M). In isolated mouse liver mitochondria, these compounds at 2–20 μM promoted oxygen consumption in a dose-dependent manner, suggesting that they act as mitochondrial uncouplers, whereas CP-DIF-1 (another derivative of DIF-1) at 10–20 μM had no effect. In confluent mouse 3T3-L1 fibroblasts, DIF-1 and DIF-1(3M) but not CP-DIF-1 induced phosphorylation (and therefore activation) of AMP kinase (AMPK) and promoted glucose consumption and metabolism. The DIF-induced glucose consumption was reduced by compound C (an AMPK inhibitor) or AMPK knock down. These data suggest that DIF-1 and DIF-1(3M) promote glucose uptake, at least in part, via an AMPK-dependent pathway in 3T3-L1 cells, whereas cellular metabolome analysis revealed that DIF-1 and DIF-1(3M) may act differently at least in part.

## 1. Introduction

**Figure 1:**
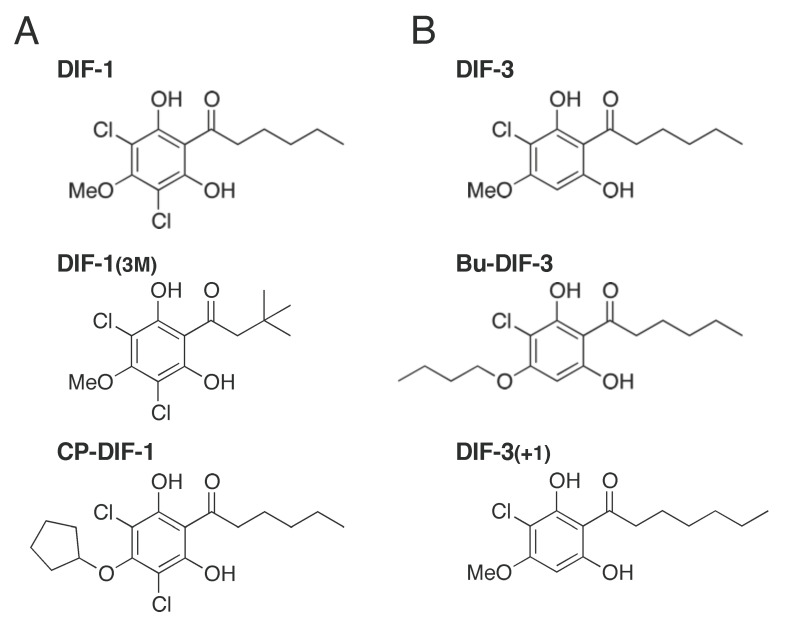
Chemical structure of differentiation-inducing factors. (**A**) DIF-1 and its derivatives used in this study. (**B**) DIF-3 and its derivatives that possess strong antitumor activities.

The cellular slime mold *Dictyostelium discoideum* is an excellent model organism for cell and developmental biology; at the end of its development, it forms fruiting bodies, each consisting of spores and a multicellular stalk [1,2]. Differentiation-inducing factor-1 (DIF-1) (Figure 1A), a chlorinated alkylphenone (a polyketide), functions as an inducer of stalk cell differentiation and also as a modulator of chemotactic cell movement in the development of *D. discoideum* [3,4,5,6]. DIF-3 (Figure 1B) is the first metabolite produced during the degradation of DIF-1 and has virtually no function in *D. discoideum* [4,5,6,7].

On the other hand, we found that DIF-3 and its derivatives such as Bu-DIF-3 and DIF-3(+1) (Figure 1B) possessed strong antitumor activities in mammalian cells, suggesting that these compounds are good leads for the development of anticancer drugs [8,9,10,11,12,13,14,15,16,17,18,19,20].

Twenty years after the discovery of DIF-1, we coincidentally found that DIF-1 can promote glucose consumption in mammalian cells, such as mouse 3T3-L1 fibroblasts (preadipocytes and a model of non-transformed cells) and 3T3-L1 adipocytes [21]. DIF-1 induces translocation of glucose transporter 1 (GLUT1) from intracellular vesicles to the plasma membrane and thereby promotes glucose uptake, at least in part, via a phosphatidylinositol 3-kinase (PI3K)/Akt-independent pathway [21]. The activity of DIF-1 and its derivative, DIF-1(3M) (Figure 1A), is more potent than that of other derivatives tested so far [21,22]. DIF-1 and DIF-1(3M) can promote glucose metabolism in 3T3-L1 cells, and oral DIF-1 administration can decrease blood glucose in streptozotocin (STZ)-treated diabetic rats [23]. These data suggest that DIF-1 and its derivatives may have therapeutic potential for the treatment of obesity and/or diabetes. However, the mechanism(s) underlying the actions of DIF derivatives remains to be elucidated.

5′-AMP-activated kinase (AMPK) is a heterotrimeric protein composed of α-, β-, and γ-subunits; when the cellular AMP-to-ATP ratio is increased by metabolic stress, the α-subunit is phosphorylated, resulting in AMPK activation [24,25,26,27]. AMPK may serve as a universal energy sensor that regulates the metabolic energy production and consumption [24,26]. Activation of AMPK stimulates the translocation of GLUT1 and GLUT4 from intracellular vesicles to the plasma membrane, promoting glucose uptake [27,28,29,30,31]. It has been shown with some cell lines that mitochondrial poisons such as azide and dinitrophenol (DNP) can activate AMPK and stimulate GLUT translocation, thereby promoting glucose uptake [32,33,34,35,36,37,38,39].

In this study, to elucidate the mechanism(s) of the glucose uptake-promoting activity of DIF-1 and DIF-1(3M), we first analyzed the effects of some DIF derivatives on mitochondrial oxygen consumption (MOC) and AMPK phosphorylation in confluent 3T3-L1 fibroblasts, and then assessed the involvement of AMPK in DIF-promoted glucose consumption by manipulating AMPK activity. We show that DIF-1 and DIF-1(3M) may promote glucose uptake, at least in part, via an AMPK-dependent pathway in 3T3-L1 cells.

## 2. Results

### 2.1. Effects of DIF Derivatives on Mitochondrial Oxygen Consumption (MOC)

**Figure 2:**
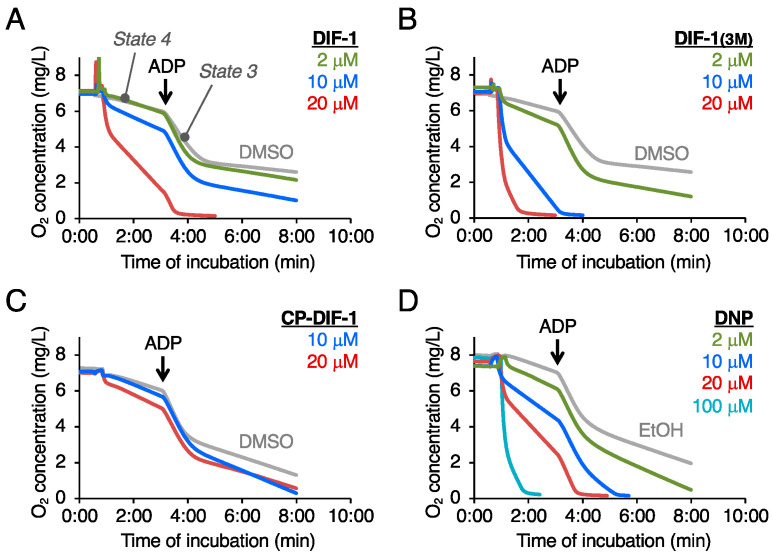
Effects of DIF derivatives and DNP on mitochondrial oxygen consumption (MOC) in isolated mouse liver mitochondria. MOC was monitored in the presence of 1% DMSO or EtOH (vehicles) or the indicated concentrations of DIF derivatives (**A**–**C**) or DNP (**D**). After recording “*State 4*” (resting) respiration, ADP (200 μM, final concentration) was added to induce “*State 3*” respiration.

We have previously shown that DIF-3 and some of its derivatives (strong antitumor agents) might function, at least in part, by promoting MOC by uncoupling mitochondrial activity [17,18]. To elucidate the mechanism of the glucose uptake-promoting activities of DIF-1 and DIF-1(3M), we analyzed the effects of DIF-1, DIF-1(3M), and CP-DIF-1 on oxygen consumption in the intact mitochondria isolated from mouse liver (Figure 2). DIF-1 at 2–20 μM promoted MOC in a dose-dependent manner (Figure 2A), as described before [18], and DIF-1(3M) was more efficient than DIF-1 (Figure 2B), whereas CP-DIF-1 at 10 or 20 μM scarcely affected MOC. As expected, the mitochondrial uncoupling agent DNP at 2–100 μM also promoted MOC in a dose-dependent manner (Figure 2C).

Hereafter, we will consider DIF-1 and DIF-3(3M) as mitochondrial uncouplers and CP-DIF-1 as a control compound.

### 2.2. Effects of DIF Derivatives on AMPK Activity in 3T3-L1 Cells

**Figure 3:**
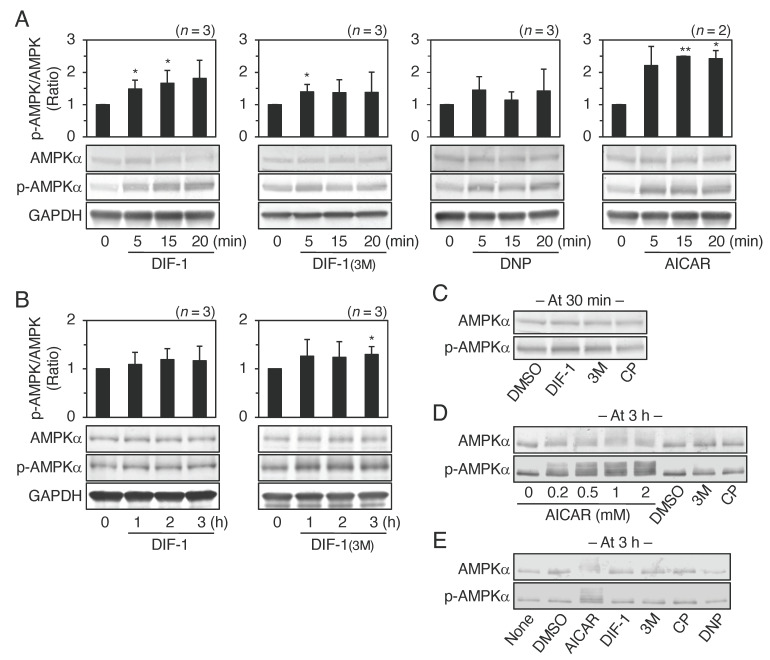
Effects of DIF-1 and DIF-1(3M) on AMPK phosphorylation in confluent 3T3-L1 cells. (**A**,**B**) Cells were incubated for the indicated times with 20 μM DIF-1 (**A**,**B**), DIF-1(3M) (**A**,**B**), 75 μM DNP (**A**), or 0.2 mM AICAR (**A**), and AMPKα and phospho-AMPKα (p-AMPKα) were analyzed by Western blotting. The graphs show the ratio of p-AMPKα/AMPKα of each sample to that of the t_0_ control; the mean values and SD of two or the three independent experiments are presented. * *p* < 0.05, ** *p* < 0.01 versus t_0_ control. (**C**) Cells were incubated for 0.5 h with 0.2% DMSO, or 20 μM DIF-1, DIF-1(3M) (3M), or CP-DIF-1 (CP), and AMPKα and p-AMPKα were analyzed by western blotting. (**D**) Cells were incubated for 3 h with the indicated concentrations of AICAR, 0.2% DMSO, or 20 μM DIF-1(3M) (3M) or CP-DIF-1 (CP), and AMPKα and p-AMPKα were analyzed by western blotting. (**E**) Cells were incubated for 3 h with or without 0.2% DMSO, 2 mM AICAR, 75 μM DNP, or 20 μM DIF derivatives, and AMPKα and p-AMPKα were analyzed by Western blotting.

Mitochondrial poisons such as DNP and azide promote glucose uptake in various cell lines via AMPK activation [32,33,34,35,36,37,38,39]. To examine the effects of DIF derivatives on AMPK activity, we assessed the phosphorylation of AMPKα by Western blotting (Figure 3). Mitochondrial uncouplers, DIF-1 and DIF-1(3M) at 20 μM significantly promoted the phosphorylation of AMPKα for 5–30 min of incubation, while the non-uncoupler, CP-DIF-1, did not (Figure 3A,C). DIF-1 and DIF-1(3M) might promote AMPKα phosphorylation after 1–3 h incubation, but the effects, if any, were small (Figure 3B,D,E). Another uncoupler, DNP at 75 μM also tended to promote AMPKα phosphorylation after 5–20 min incubation (Figure 3A). As expected [30,38,39,40], the AMPK activator AICAR, at 0.2–2 mM greatly promoted and maintained AMPKα phosphorylation for a relatively long time of incubation (Figure 3A,D,E).

### 2.3. Effects of DIF Derivatives, DNP, and AICAR on Glucose Consumption in 3T3-L1 Cells

**Figure 4:**
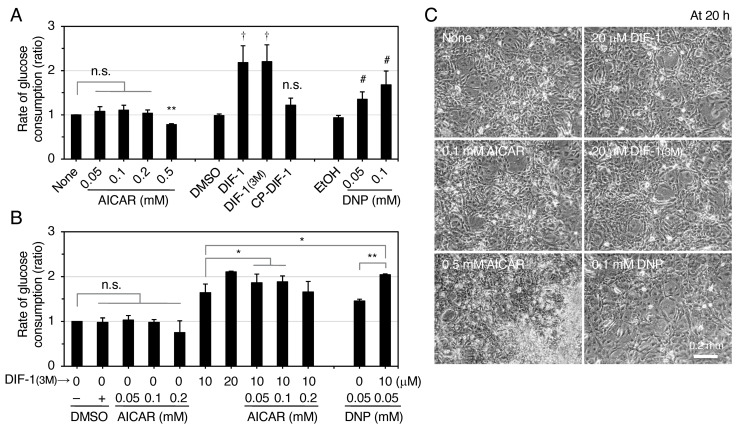
Effects of DIF derivatives on glucose consumption in confluent 3T3-L1 cells. (**A**) Cells were incubated for 15–20 h in the presence or absence of 0.2% DMSO or EtOH (vehicles), 20 μM DIF derivatives or the indicated concentrations of AICAR and DNP. The glucose concentration in each medium was measured, and the rate of glucose consumption was calculated. The mean values and SD of the three independent experiments are presented. ** *p* < 0.01, ^†^ *p* < 0.05, ^#^ *p* < 0.05 versus control; n.s., not significant. (**B**) Cells were incubated for 15–20 h in the presence of the indicated additives and the combined effects of DIF-1(3M) and AICAR or DNP on glucose consumption were also assessed as in (**A**). The mean values and SD of the four independent experiments are presented. * *p* < 0.05, ** *p* < 0.01; n.s., not significant. (**C**) Cells were incubated for 20 h in the presence or absence of the indicated concentrations of the additives and observed by a phase-contrast microscope.

To assess the relationship between AMPK activation and glucose uptake (consumption) induced by the compounds, we analyzed the effects of DIF derivatives, DNP, and AICAR on glucose consumption (Figure 4). As expected, DIF-1 and DIF-1(3M) at 20 μM promoted glucose consumption by more than 2-fold [21,22], while CP-DIF-1 at 20 μM did not (Figure 4A). DNP at 0.05 or 0.1 mM promoted glucose consumption by 1.3–1.7-fold (Figure 4A). DIF derivatives at 20 μM and DNP at 0.1 mM were not toxic to the cells (Figure 4C). AICAR at 0.05–0.2 mM did not significantly promote glucose consumption (Figure 4A,B) and was toxic to the cells at 0.5 mM (Figure 4C), although it strongly activated AMPK at 0.2–2 mM (Figure 3D). Since AICAR can activate many other AMP-dependent enzymes [41], long-term stimulation (15–20 h) with AICAR might disturb some cell function and thus cause toxicity to 3T3-L1 cells. AMPK activation by DIF derivatives or DNP was much weaker than that by AICAR (Figure 3). Taken together, these results suggest that the involvement of AMPK activation, if any, in the actions of DIF-1 and DIF-1(3M) may be partial.

### 2.4. Effects of AMPK Inhibition on DIF-Promoted Glucose Uptake in 3T3-L1 Cells

**Figure 5:**
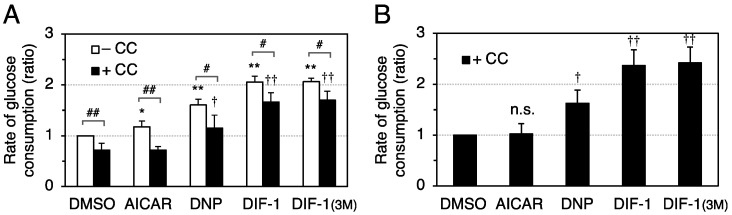
Effects of compound C on DIF-induced glucose consumption in confluent 3T3-L1 cells. Cells were pre-incubated for 0.5 h with 15 μM compound C (CC) and further incubated for 8–15 h in the presence of DMSO (0.3%), AICAR (0.1 mM), DNP (50 μM), DIF-1 (15 μM), or DIF-1(3M) (15 μM). The glucose concentration of each medium was measured, and the rates of glucose consumption are shown relative to the DMSO control (–CC) in (**A**) and to the DMSO control (+CC) in (**B**). The mean values and SD of the four independent experiments are presented. * *p* < 0.05, ** *p* < 0.01 versus DMSO control (–CC); ^†^ *p* < 0.05, ^††^ *p* < 0.01 versus DMSO control (+CC); ^#^ *p* < 0.05, ^##^ *p* < 0.01; n.s., not significant.

We then examined the effects of compound C, an inhibitor of AMPK, on glucose uptake in the presence of AICAR, DNP, DIF-1, or DIF-1(3M) (Figure 5). In DMSO control cells, 15 μM compound C reduced the basal rate of glucose consumption by 20%. AICAR at 0.1 mM slightly but significantly promoted glucose consumption, and this effect was completely inhibited in the presence of compound C. Glucose consumption was significantly increased by DNP at 50 μM (1.5-fold) and by DIF-1 or DIF-1_(3M)_ at 15 μM (2-fold).

**Figure 6:**
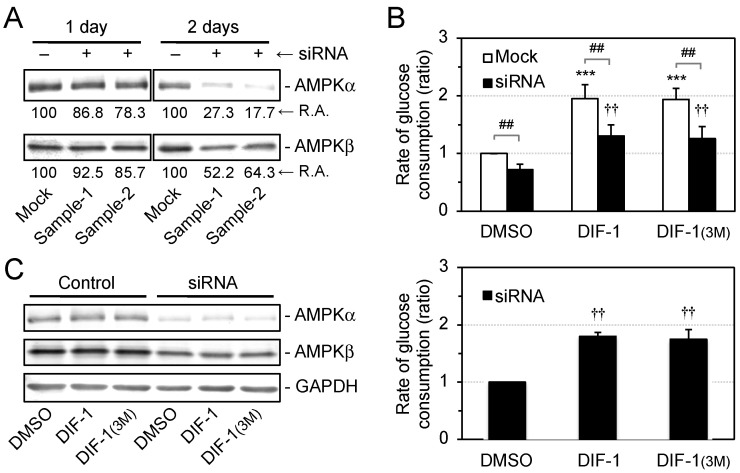
Effects of AMPK knockdown on DIF-induced glucose consumption in confluent 3T3-L1 cells. (**A**) RNAi was performed once (1 day) or twice (2 days) with a mock or siRNA against AMPKα in duplicate (Sample-1 and -2). AMPKα and AMPKβ were analyzed by Western blotting, and their relative amounts (R.A.) were assessed. AMPKα was knocked down efficiently after 2-day RNAi. (**B**) Cells with AMPKα knocked down for 2 days were incubated for 10–20 h with DMSO (0.2%), DIF-1 (20 μM), or DIF-1(3M) (20 μM). The glucose concentration of each medium was measured, and the rates of glucose consumption are shown relative to the DMSO control (mock) in the top graph and to the DMSO control (RNAi) in the bottom graph. The mean values and SD of the four independent experiments are presented. *** *p* < 0.001 versus DMSO control (mock); ^††^ *p* < 0.01 versus DMSO control (RNAi); ^##^ *p* < 0.01. (**C**) After the measurements shown in (B), AMPKα, AMPKβ, and GAPDH in the same cells were analyzed by Western blotting. AMPKα was knocked down efficiently.

We then examined the effects of AMPKα knockdown on glucose consumption in the presence of DIF-1 or DIF-1(3M) (Figure 6). Transfection with siRNA against AMPKα performed twice decreased the AMPKα protein level by 80% in confluent 3T3-L1 cells (Figure 6A) and significantly reduced the rate of glucose consumption in the presence of 0.2% DMSO or 20 μM DIF-1 or DIF-1(3M) (Figure 6B). Importantly, however, both DIF-1 and DIF-1(3M) significantly increased the rate of glucose consumption (1.8-fold) under the conditions (Figure 6B) despite AMPKα knockdown throughout the assay (Figure 6C).

These results suggest that DIFs promote glucose uptake via an AMPK-dependent pathway, at least in part. On the other hand, DIFs may function via an AMP-independent pathway in parallel with the AMPK-dependent pathway, although we cannot exclude that DIFs promoted glucose consumption via a very small amount of remaining AMPK activity in the presence of compound C (Figure 5) or AMPK protein under the AMPK knockdown conditions (Figure 6).

### 2.5. Effects of DIF Derivatives on Glucose Metabolism in 3T3-L1 Cells

**Figure 7:**
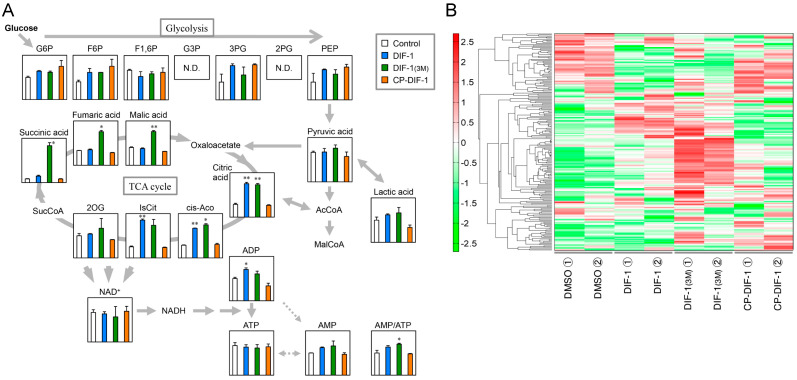
Metabolome analysis. (**A**) Effects of DIFs on glucose metabolism in confluent 3T3-L1 cells. Cells were incubated for 3 h with 0.1% DMSO (Control) or 20 μM of DIF-1, DIF-1(3M), or CP-DIF-1, and metabolite levels per 10^6^ cells were determined by use of CE-TOFMS to construct a metabolome pathway map. The metabolites levels in the control cells were set to 1, and the relative amounts in the DIF-treated cells are presented as the mean and SD of the duplicate samples. * *p* < 0.05 versus Control. N.D., not detected; G6P, glucose 6-phosphate; F6P, fructose 6-phosphate; F1,6P, fructose 1,6-diphosphate; G3P, glyceraldehyde 3-phosphate; 3PG, 3-phosphoglyceric acid; 2PG, 2-phosphoglyceric acid; PEP, phosphoenolpyruvic acid; AcCoA, acetyl CoA_divalent; MalCoA, malonyl CoA_divalent; cis-Aco, cis-aconitic acid; IsCit, isocitric acid; 2OG, 2-oxoglutaric acid. (**B**) Heat map of cellular metabolites. Of the 205 metabolite peaks identified in this study, metabolites showing similar relative abundance throughout the 4 duplicated samples were clustered into a metabolome heat map. The horizontal axis shows the sample names, and the vertical axis shows the metabolites. HCA (hierarchical cluster analysis) was performed, and the distance between the peaks is shown in the dendrogram. Green, small average abundance; red, large average abundance.

Using CE-TOFMS (capillary electrophoresis time-of-flight mass spectrometry), we performed a metabolome analysis and showed that DIF-1 and DIF-1(3M) promoted glucose metabolism but did not significantly affect cellular ATP level in 3T3-L1 cells [23]. In this study, to further assess the differences, if any, in the effects of the DIF derivatives, we used the same approach to analyze the effects of 20 μM DIF-1, DIF-1(3M), and CP-DIF-1 on glucose metabolism and the AMP/ATP ratio (Figure 7A). DIF-1 and DIF-1(3M) tended to increase the glucose metabolite levels but did not significantly affect cellular ATP level (Figure 7A). DIF-1(3M) increased the AMP/ATP ratio slightly but significantly, while DIF-1 tended to increase it (Figure 7A). At the same concentration, CP-DIF-1 did not significantly affect glucose metabolite levels, ATP level, or the AMP/ATP ratio (Figure 7A).

A heat map of cellular metabolites showed considerable differences between cells incubated with DIF-1 or DIF-1(3M) and DMSO control cells, while CP-DIF-1 slightly affected the metabolites in comparison with the DMSO control (Figure 7B). The effects of DIF-1 and DIF-1(3M) on cellular metabolites differed considerably from each other (Figure 3A and Figure 4). These results suggest that the mechanisms underlying the actions of the two DIFs differ from each other, at least in part.

## 3. Discussion

### 3.1. DIF Derivatives That Promote Glucose Consumption (Uptake) in Mammalian Cells

As already described, DIF-1 (Figure 1A) is a differentiation-inducing factor and chemotaxis modulator in *D. discoideum* [3,4,5,6], while DIF-3 (Figure 1B) is the first metabolite produced during the degradation of DIF-1 [4,5,6,7].

In the 1990s, we showed that DIF-1, DIF-3, and their derivatives possess antitumor activities in mammalian cells [8,9,10,11,12,13,14,15,16,17,18,19,20]. DIF-3 and its derivatives possess more potent antitumor activities than DIF-1 and its derivatives in several tumor cell lines such as human leukemia K562 cells and human cervical cancer HeLa cells [12,14,16,17,18,19,42]. DIF-3 derivatives such as Bu-DIF-3 and DIF-3(+1) (Figure 1B) are promising lead compounds for anticancer agents [16,17,18,19,42,43], and thus DIF-1 and DIF-1(3M) are no longer at the center of our anticancer drug research. On the other hand, we found that DIF-1 and DIF-1(3M) are promising leads for the development of antiobesity and antidiabetes drugs that possess strong glucose uptake-promoting activities in mammalian cells in vitro [19,21,22].

In the light of this special issue “Cancer Biology in Diabetes”, it should be noted that AMPK activators such as AICAR and metformin can inhibit tumor cell growth [40,44,45,46,47], and metformin has been used in some clinical trials [45,48]. Anticancer and antidiabetes agents such as DIF derivatives may have some common mechanisms of action, which we intend to investigate in the future.

### 3.2. Involvement of AMPK in the Actions of DIF-1 and DIF-1(3M)

DIF-1 triggers GLUT1 translocation from an intracellular pool to the plasma membrane via a PI3K/Akt-independent pathway, thus promoting glucose uptake in both 3T3-L1 fibroblasts and 3T3-L1 adipocytes [21]. DIF-1 and DIF-1(3M) also promote the metabolism of glucose taken up by the cells [23].

**Figure 8:**
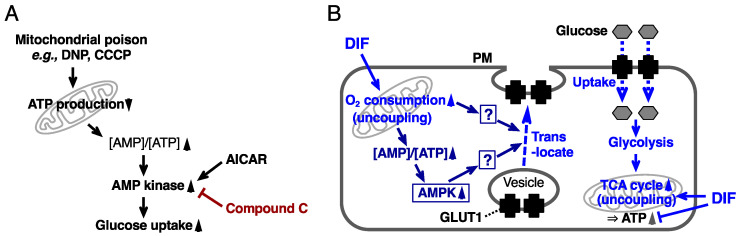
(**A**) Scheme of the actions of mitochondrial poisons such as DNP and CCCP. These compounds reduce ATP production and thus increase the AMP/ATP ratio, activating AMP kinase and promoting glucose uptake. AICAR is an activator and compound C is an inhibitor of AMP kinase. (**B**) Proposed scheme for the actions of DIF. By uncoupling mitochondrial activities, DIF may disturb ATP production and activate AMPK, which may then induce GLUT1 translocation to the plasma membrane and glucose uptake. It is generally unknown how AMPK activation induces GLUT1 translocation, whereas AMPK activation may inhibit GLUT1 internalization (endocytosis) to promote glucose uptake by triggering the degradation of TXNIP (thioredoxin-interacting protein), a stimulator of GLUT1 endocytosis [49,50]. Note that DIF may induce GLUT1 translocation partly via an AMPK-independent pathway, but it remains to be elucidated how DIF induces GLUT1 translocation. Glucose may be metabolized immediately via glycolysis and via the TCA cycle.

Since mitochondrial uncouplers have been shown to promote glucose uptake by activating AMPK [32,34,35,36,37], in the present study we assessed the involvement of AMPK in DIF-induced glucose uptake in 3T3-L1 cells, comparing the effects of DIF-1, DIF-1(3M), and CP-DIF-1. We showed here that (1) DIF-1 and DIF-1(3M) but not CP-DIF-1 promoted MOC (Figure 2), (2) DIF-1 and DIF-1(3M) but not CP-DIF-1 induced the phosphorylation (and therefore activation) of AMPKα (Figure 3), (3) DIF-1 and DIF-1(3M) but not CP-DIF-1 promoted glucose uptake (Figure 4), and (4) suppression of AMPK activity significantly reduced the glucose uptake induced by DIF-1 and DIF-1(3M) (Figure 5 and Figure 6). These results suggest that DIF-1 and DIF-1(3M) promote glucose uptake by mitochondrial uncoupling and subsequent activation of AMPK, at least in part (Figure 8). However, since neither compound C nor AMPK knockdown completely inhibited DIF-induced glucose uptake (Figure 5 and Figure 6) and also because the AMPK activator, AICAR (Figure 3), did not promote (Figure 4A) or only slightly promoted glucose uptake (Figure 5A), DIF-1 and DIF-1(3M) may also promote glucose uptake via an AMPK-independent pathway (Figure 8B).

In this study, we analyzed the metabolic pathway of glucose in the presence of three DIF derivatives and found that the mitochondrial uncouplers DIF-1 and DIF-1(3M) but not the non-uncoupler CP-DIF-1 promoted glucose metabolism without affecting the cellular ATP level (Figure 7A); the DIF-1 and DIF-1(3M) data agree well with our previous results [23]. A slight increase in the AMP/ATP ratio by DIF-1 and DIF-1(3M) (Figure 7A) might activate AMPK (Figure 3).

We also revealed that the metabolomes of cells treated with DIF-1 or DIF-1(3M) differed from each other (Figure 7B), suggesting that the two compounds promote glucose consumption via different pathways, at least in part. We will further elucidate the precise mechanisms underlying the actions of DIF-1 and DIF-1(3M) (i.e., the blank part of the scheme in Figure 8B) and try to develop novel antiobesity and antidiabetes agents on the basis of these compounds.

## 4. Materials and Methods

### 4.1. Cells and Reagents

Mouse 3T3-L1 fibroblast cells were used in this study; 3T3-L1 cells were maintained in vitro at 37 °C (5% CO2) in DMEM-HG (Dulbecco’s Modified Eagle’s Medium containing a high concentration (4,500 mg/L) of glucose (D5796; Sigma, St. Louis, MO, USA) supplemented with 75 μg/mL penicillin, 50 μg/mL streptomycin and 10% (*v*/*v*) fetal bovine serum (FBS)). DIF-1, DIF-1(3M), and CP-DIF-1 were synthesized as previously described [16] and stored at –20 °C as 2.5–10 mM solutions in dimethylsulfoxide (DMSO). DNP, 5-aminoimidazole-4-carboxamide-1-β-d-ribofuranoside (AICAR), and 6-[4-(2-Piperidin-1-ylethoxy) phenyl]-3-pyridin-4-ylpyrazolo [1,5-a] pyrimidine (compound C) were obtained from Wako Pure Chemical Industries (Osaka, Japan). DNP were dissolved in ethanol (EtOH) and compound C in DMSO. Rabbit antibodies against AMPKα, phospho-AMPKα, AMPKβ, phospho-AMPKβ, and glyceraldehyde 3-phosphate dehydrogenase (GAPDH) were purchased from Cell Signaling Technology (Beverly, MA, USA). Alkaline phosphatase-conjugated goat anti-rabbit IgG antibody, nitroblue tetrazolium (NBT), and 5-bromo-4-chloro-3′-indolylphosphate (BCIP) were purchased from Promega (Madison, WI, USA). The small interfering RNA (siRNA) for AMPKα1/2 (sc-45313) was obtained from Santa Cruz Biotechnology Inc. (Dallas, TX, USA).

### 4.2. Measurement of Mitochondrial Oxygen Consumption

Mitochondria were isolated from mouse liver (ICR; 7–10-week-old females) by differential centrifugation as described previously [17,51]. Mitochondrial oxygen consumption was measured using a Clark-type oxygen electrode (Strathkelvin Instruments Ltd., North Lanarkshire, Scotland, UK) as described previously [52,53]. Briefly, the mitochondria-enriched fraction was incubated at 30 °C in an oxygen measurement buffer (225 mM mannitol, 75 mM sucrose, 5 mM succinate, 5 mM glutamate, 10 mM KCl, 0.1 mM EDTA, 3 mM phosphate, and 20 mM Tris-HCl, pH 7.4) in the presence of a vehicle (DMSO or EtOH) or various concentrations of DIF-1 and its derivatives. After recording “State 4” (resting) respiration reaction, an aliquot of ADP was added to a final concentration of 200 μM to induce “State 3” respiration reaction [17,51].

### 4.3. Assessment of Glucose Consumption (Uptake) in 3T3-L1 Cells

The rate of glucose consumption was assessed mostly as described previously [21]. Cells were incubated in DMEM-HG (1 mL/well, 12-well plates) for 3–5 days until they reached the confluency; DMEM-HG was exchanged every 2 days. The cells were then preincubated for 1–2 days with 1 mL of DMEM-MG (DMEM containing a medium concentration (2000 mg/L) of glucose supplemented with the antibiotics, 10% FBS, and 10 mM HEPES-NaOH (pH 7.4)). The cells were then treated for 8–20 h with 1 mL of fresh DMEM-MG containing the additives. Glucose concentration in the aliquots of the incubation media was determined using a blood glucose meter and appropriate sensor chips (Sanwa Chemical Institute, Osaka, Japan). The approximate rate of glucose consumption was then calculated. Note that the rate of glucose consumption measured by the above procedure matches well that of glucose uptake assessed with 2-[1,2-3*H*]deoxy-d-glucose [21]; therefore, sometimes we refer to the rate glucose consumption as “rate of glucose uptake”.

### 4.4. Western Blotting

Confluent 3T3-L1 cells were incubated in DMEM-MG (1 mL/well in a 12-well plate) containing the additives; incubation times are indicated in the figures. Cells were washed with 1 mL/well of PBS (20 mM phosphate buffered saline, pH 7.4), harvested, and lysed by adding an SDS (sodium dodecyl sulfate)-sample buffer (200 μL/well), destroyed and heated by sonication, and used for SDS-PAGE. Protein transfer and immunoblotting were performed as described previously [17], by using a primary antibody for AMPKα, phospho-AMPKα, AMPKβ, phospho-AMPKβ, or GAPDH, and a second antibody, an alkaline phosphatase-conjugated anti-rabbit IgG antibody. Color development (visualization of the protein bands) was performed in an alkaline buffer (100 mM Tris-HCl, pH 9.5, 100 mM NaCl, and 5 mM MgCl_2_) containing NBT (125 μg/mL) and BCIP (62.5 μg/mL). Visualized protein bands were then digitized and quantified by using Adobe Photoshop CS4 (version 11.0) (Adobe, San Jose, CA, USA) and ImageJ Software (version 1.53) (http://imagej.nih.gov/ij/ (accessed on 25 February 2021)).

### 4.5. RNA Interference (RNAi) Using Small Interfering RNA (siRNA)

RNAi was performed according to the manufacturer’s instructions (Invitrogen; ThermoFisher Scientific, Waltham, MA, USA), except that we used confluent cells. The 3T3-L1 cells grown in DMEM-HG (1 mL/well in a 12-well plate) were washed with PBS (1 mL/well), and DMEM-HG without the antibiotics (DMEM-HG(–Ab)) was added (1 mL/well). Then, the first RNAi was performed. Mock solution containing Lipofectamine RNAiMAX reagent (3 μL) and Opti-MEM I medium (97 μL) (Invitrogen) or siRNA solution containing Lipofectamine RNAiMAX reagent (3 μL), Opti-MEM I medium (94 μL), and AMPKα1/2 siRNA solution (3 μL of 10 μM solution) was each added to three wells. After 24 h, the cells were washed with PBS (1 mL/well), then incubated in fresh DMEM-HG(—Ab)(1 mL/well), and the second RNAi was performed by adding 100 μL of the same mock and siRNA solutions to the wells. After 24 h, the media were removed, the cells were washed with PBS (1 mL/well) and used for Western blotting to check AMPKα expression (and AMPKβ and GAPDH expression for comparison). For glucose consumption assay, the media used for RNAi were replaced with DMEM-MG (1 mL/well), and the cells were incubated for 2 h. Then, the cells were incubated for 8–15 h with fresh DMEM-MG (1 mL/well) containing 0.2% (*v*/*v*) DMSO, 20 μM DIF-1, or 20 μM DIF-1(3M), and glucose consumption was assessed as described in Section 4.3. After the glucose consumption assay, the cells were used for Western blotting to check AMPKα expression (and AMPKβ and GAPDH expression for comparison) again.

### 4.6. Metabolome Analysis

Confluent 3T3-L1 cells in 90-mm tissue culture dishes were incubated for 3 h with 10 mL of DMEM-MG containing 0.1% (*v*/*v*) DMSO or 20 μM DIF-1, DIF-1(3M), or CP-DIF-1; the assay was performed in duplicate. The culture media were removed, and the cells were washed with 10 mL per dish of 5% (*w*/*v*) mannitol solution and then 2 mL of the same solution. The cells were collected by scraping in methanol (1.3 mL/well) containing 10 μM internal standard solution (Human Metabolome Technologies, Tokyo, Japan) and transferred into eight centrifugation tubes. Ionic metabolites were analyzed by capillary electrophoresis time-of-flight mass spectrometry (Agilent CE-TOFMS system; Agilent Technologies, Waldbronn, Germany) as described previously [23,54,55,56,57]. Relative quantification data for the identified metabolites were used for hierarchical cluster analysis (HCA) and principal component analysis (PCA) performed with the proprietary software, PeakStat and SampleStat (Human Metabolome Technologies), respectively, to produce a metabolome heat map and a metabolome pathway-map.

### 4.7. Statistical Analysis

Welch’s *t*-test was used for the statistical analyses. Values were considered to be significantly different when the *p* value was less than 0.05.

## 5. Patents

The following authors hold a patent related to this article:

Kubohara, Y.; Shibata, H. Method of lowering blood glucose and method of treating diabetes and obesity. Japanese Patent No. 4534039, 25 June 2010.

## Data Availability

Not applicable.
